# Near-infrared photoimmunotherapy: design and potential applications for cancer treatment and beyond

**DOI:** 10.7150/thno.74820

**Published:** 2022-10-09

**Authors:** Danfeng Wei, Jinxin Qi, Michael R Hamblin, Xiang Wen, Xian Jiang, Hao Yang

**Affiliations:** 1 Department of Dermatology and National Clinical Research Center for Geriatrics, West China Hospital, Sichuan University, Chengdu 610041, China.; 2 Laboratory of Dermatology, Clinical Institute of Inflammation and Immunology, Frontiers Science Center for Disease-related Molecular Network West China Hospital, Sichuan University, Chengdu 610041, China.; 3 NHC Key Lab of Transplant Engineering and Immunology, Organ Transplant Center, West China Hospital, Sichuan University, Chengdu, Chengdu 610041, China.; 4 Laser Research Centre, Faculty of Health Science, University of Johannesburg, Doornfontein 2028, South Africa.; 5 Sichuan Provincial Engineering Laboratory of Pathology in Clinical Application, West China Hospital, Sichuan University.

**Keywords:** near-infrared photoimmunotherapy, IRdye 700DX, target-specific photosensitizer conjugate, cancer-treatment, noncancerous diseases

## Abstract

Near-infrared photoimmunotherapy (NIR-PIT) is a newly developed cancer treatment modality based on a target-specific photosensitizer conjugate (TSPC) composed of an NIR phthalocyanine photosensitizer and an antigen-specific recognition system. NIR-PIT has predominantly been used for targeted therapy of tumors via local irradiation with NIR light, following binding of TSPC to antigen-expressing cells. Physical stress-induced membrane damage is thought to be a major mechanism underlying NIR-PIT-triggered photokilling. Notably, NIR-PIT can rapidly induce immunogenic cell death and activate the adaptive immune response, thereby enabling its combination with immune checkpoint inhibitors. Furthermore, NIR-PIT-triggered “super-enhanced permeability and retention” effects can enhance drug delivery into tumors. Supported by its potential efficacy and safety, NIR-PIT is a rapidly developing therapeutic option for various cancers. Hence, this review seeks to provide an update on the (i) broad range of target molecules suitable for NIR-PIT, (ii) various types of receptor-selective ligands for designing the TSPC “magic bullet,” (iii) NIR light parameters, and (iv) strategies for enhancing the efficacy of NIR-PIT. Moreover, we review the potential application of NIR-PIT, including the specific design and efficacy in 19 different cancer types, and its clinical studies. Finally, we summarize possible NIR-PIT applications in noncancerous conditions, including infection, pain, itching, metabolic disease, autoimmune disease, and tissue engineering.

## Introduction

Owing to its high morbidity and mortality, cancer remains a great threat to human health despite the enormous amount of associated research. Photodynamic therapy (PDT) is a promising treatment modality as it offers efficient antitumor destruction with limited adverse effects via localized activation of a photosensitizer (PS) by light irradiation. PDT depletes cancer cells through reactive oxygen species generation or physical stress, consequently inducing cellular apoptosis or necrosis 1. Although PDT has shown many promising results, its clinical application is limited by poor PS solubility and lack of targeted delivery methods for most PSs.

**Figure 1:**
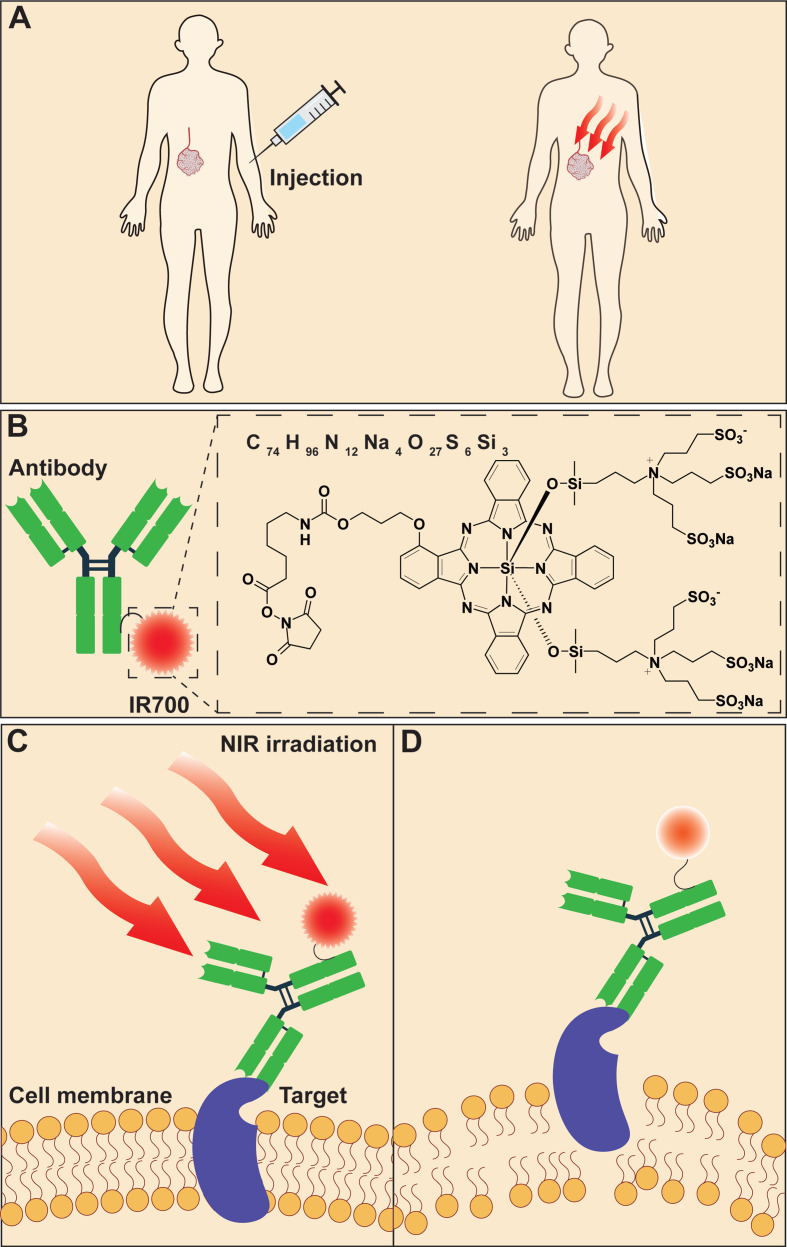
Scheme of NIR-PIT based on TSPCs. (A) The systematically injected TSPCs can be locally activated following NIR irradiation at lesion sites. (B) Schematic illustration of TSPC. (C) TSPCs bind to specific target molecules on the cell surface followed by NIR irradiation. (D) Photoinduced dissociation of hydrophilic axial ligands from IR700 causes transmembrane damage through the remaining hydrophobic complexes on the cell membrane. NIR-PIT: near-infrared photoimmunotherapy; TSPC: target-specific photosensitizer conjugate.

A newly developed molecular-targeted antitumor phototherapy approach, called near-infrared photoimmunotherapy (NIR-PIT), has shown great potential in overcoming the challenges associated with PDT. NIR-PIT employs a tumor-specific binding ligand, such as a monoclonal antibody (mAb), mAb fragment, or small-molecule ligand that is conjugated to IRdye 700DX (IR700)—a silicon phthalocyanine dye—which is activated by NIR light to cause immunogenic cell death (ICD) 2, 3. After *in vivo* administration, the target-specific photosensitizer conjugate (TSPC) distributes into the tumor and binds to the antigen-expressing cell membranes (Figure 1A-B). Following topical NIR light irradiation at the tumor site, photoinduced dissociation of hydrophilic axial ligands from IR700 leads to the aggregation of hydrophobic complexes on the cell membrane, which causes transmembrane damage and reduces cell membrane integrity (Figure 1C-D). The tumor cells then swell and burst, releasing intracellular contents due to the influx of water. As a result, NIR-PIT induces TSPC-bound tumor cell necrosis 4-6. The rapid necrotic death of cancer cells results in release of damage-associated molecular patterns (DAMPs), including calreticulin, heat shock proteins (HSPs), ATP, HMGB1, and other antigens, which altogether activate dendritic cells (DCs) to stimulate the infiltration of CD8^+^ cytotoxic T lymphocytes (CTLs). The activated T cells then elicit a host immune response against residual tumor cells following direct killing by NIR-PIT. This is the process of NIR-PIT-induced ICD 3, 7-9. In contrast, the normal bystander cells expressing minimal, or no, target antigens are spared. The remarkable cytotoxicity and specificity for tumors make NIR-PIT an attractive therapeutic modality that differs from conventional PDT. In fact, NIR-PIT-triggered ICD has the potential to augment the efficacy of various cancer immunotherapies, which will be discussed later in a subsection.

PS is a critical component of the “magic bullet” TSPC for NIR-triggered cytotoxicity. Among several PSs that have been investigated, the new-generation dye IR700 (Figure 1B) has many features that make it suitable for cancer treatment. First, the chemical modification-yielding hydrophilic feature makes IR700 superior for TSPC preparation and application compared to most conventional PSs that are typically hydrophobic. Moreover, its water-soluble property prevents IR700 aggregation or precipitation during the preparation of TSPC in buffered physiological solution, even at high dye-to-protein ratios. Upon intravenous injection, hydrophilic IR700 does not affect the tumor-homing ability or tissue distribution of antibodies, whereas hydrophobic PS-antibody conjugates may aggregate and accumulate in the liver where they are metabolized 2, 6, 7. In addition, the hydrophilic property of IR700 prevents interactions with non-antigen-expressing cell membranes, leading to little nonspecific cytotoxicity. Second, NIR light irradiation can cleave the axial ligands—consisting of three sulfonic acids as water-soluble protecting groups—of the IR700 structure, resulting in TSPC-bound membrane destruction and cell necrosis 4, 6. Thus, it is not necessary for IR700-based TSPCs to be internalized by cells, nor do they necessarily rely on reactive oxygen species for their anticancer activity 10. In contrast, conventional PSs, which are generally hydrophobic, must be internalized by targeted cells, thus, hindering their target selectivity and antitumor efficacy 1, 11. Third, IR700 molecules can be modified with an N-hydroxysuccinimide ester (NHS) group, which facilitates conjugation with antibodies or other tumor-homing species. Fourth, IR700 is activated by NIR light (690 nm), which allows for relatively deep tissue penetration. Conventional PSs typically absorb visible light with less penetration into deep tissues 2, 12, 13. Fifth, similar to other phthalocyanine PSs, IR700 has a large molar extinction coefficient, moderate quantum yield, and relatively good photochemical stability under natural light 2, 14. Sixth, IR700 can emit near-infrared fluorescence, enabling the fluorescent imaging of targeted tissues, which may facilitate more specific tumor irradiation. Moreover, a commercially-available indocyanine green-camera can monitor the therapeutic progress in real-time with decay of the IR700 fluorescence signal even during therapeutic exposure to NIR light 15, 16.

**Figure 2:**
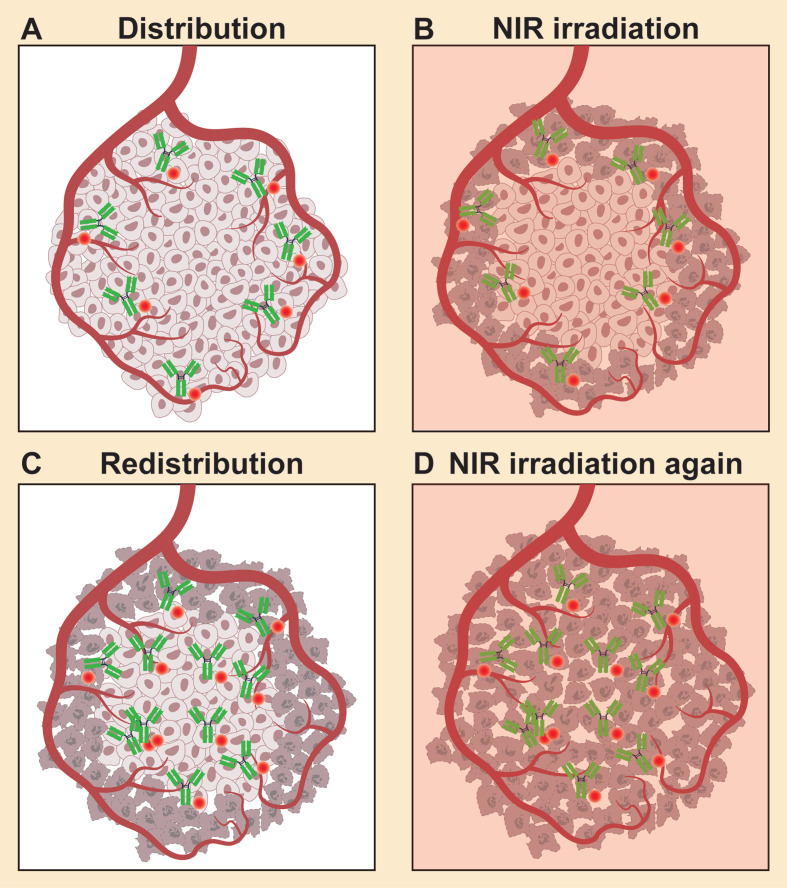
SUPR effects induced by NIR-PIT. (A) Following injection, TSPCs are delivered through the vasculature and then bind to the tumor sites. (B) Tumor necrosis following NIR irradiation. (C) Super enhanced delivery of TSPCs into the treated tumor bed after the first NIR-PIT. (D) Repeated NIR irradiation enhances the therapeutic effect of NIR-PIT. SUPR: super enhanced permeability and retention.

An effect called “super-enhanced permeability and retention” (SUPR) was observed after NIR-PIT. As NIR-PIT rapidly kills perivascular cancer cells, tumor vascular permeability greatly increases, leading to much higher (up to 24-fold) nanoparticle accumulation within the treated tumor 17. Hence, nanoparticle-based drugs can show an improved antitumor effect with the help of NIR-PIT 18. SUPR effects also facilitate the delivery of TSPC itself more deeply into the tumor after the initial NIR-PIT; repeated NIR exposures result in a better therapeutic response (Figure 2) 19-21. In theory, repeated NIR-PIT should have no safety concerns 3, 4, 19. That is, NIR light has almost no limitations on the cumulative irradiation dosage, as it is nonionizing and harmless to DNA or normal cells. IR700, whether intact or catabolized, is readily excreted into the urine without any long-term photosensitizing effects 2.

The ability of NIR-PIT to induce SUPR effects and trigger anticancer host immune responses has previously been reviewed. Its application in the local control of solid cancers, as well as certain molecular targets, antitumor mechanisms, and possible challenges in its implementation, have recently been discussed 3, 8, 22-25. Here, in the first section, we provide an update on the broad spectrum of target molecules used for NIR-PIT, various types of receptor-selective ligands for the design of a TSPC “magic bullet” (Tables 1-4), NIR light parameters, and potential strategies for enhancing the antitumor effects of NIR-PIT. In the second section, we provide a comprehensive overview of NIR-PIT applications for treating 19 different types of cancers, arising from different human tissues and organs. Meanwhile, we summarize the clinical trials on NIR-PIT for tumor treatment. In the third section, we prospectively review the possible applications of NIR-PIT in non-malignant diseases, such as infection, pain, itching, metabolic diseases, autoimmune diseases, and tissue engineering, as well as the limitations and challenges encountered when employing NIR-PIT.

## Selection of therapeutic targets

### The expression level of therapeutic targets in tumors

The accurate selection of molecules to be targeted by ligands is critical for cancer therapy. For most cancer-targeted therapies, significantly higher expression of the target molecules on cancer cells relative to that on normal cells is crucial for reducing unwanted off-target effects. However, in the case of NIR-PIT, researchers have primarily considered the distribution and function of the targeted antigens as NIR irradiation can be spatially confined to the tumor, and normal tissue is generally not damaged when using TSPC-delivered PS. Therefore, NIR-PIT has a wider target pool than other methods using other payloads that do not require external activation.

**Figure 3:**
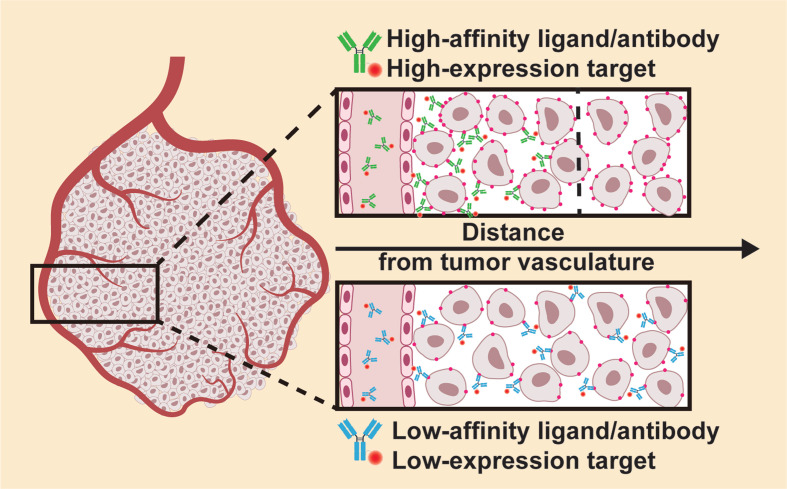
Binding site barrier of TSPC in a tumor. The preferential accumulation of high-affinity ligands/antibodies in the immediate proximity of blood vessels might hinder the deep penetration of TSPCs into the tumor parenchyma.

Although the expression level of target molecules typically does not cause safety concerns in NIR-PIT, it does impact the therapeutic effect. That is, a higher expression level of the target will induce a more potent antitumor effect as more TSPCs can bind to the target and accumulate in the tumor site. However, using a high-affinity ligand/antibody recognizing a highly-expressed target molecule may not always prove beneficial. Deep penetration into the tumor parenchyma may be hampered by accumulation of high-affinity binding ligands/antibodies in the immediate proximity of blood vessels, a phenomenon known as the “binding site barrier” (Figure 3) 26. Therefore, a more homogeneous distribution of TSPCs in the tumor parenchyma could be achieved by using an additional antibody with low antigen affinity, or an antibody that recognizes a low-expression antigen. Nakajima et al*.* developed a TSPC by employing the anti-epidermal growth factor receptor (EGFR) antibody panitumumab (Pan) plus the anti-CD25 antibody basiliximab (Bas) for targeted delivery of IR700 to the ATAC4 cell line, with high expression of EGFR and low expression of CD25. No significant difference was observed in the therapeutic response to NIR-PIT using Pan-IR700 or the Pan-IR700/Bas-IR700 mixture *in vitro*. However, the cocktail of Pan-IR700 + Bas-IR700 resulted in greater inhibition of tumor growth and longer survival in tumor-bearing mice than either Pan-IR700 or Bas-IR700 alone. Analysis of the TSPC microdistribution in the tumors showed that Pan-IR700 was more peripherally localized despite the high expression of EGFR on tumor cells, whereas Bas-IR700, targeting the low-expression CD25, was distributed uniformly throughout the tumor, including the center where Pan-IR700 was not found 27.

### Therapeutic targets on cancer cells

Despite a wide range of options, due to their overexpression and wide distribution in multiple cancers, EGFR and human epidermal growth factor receptor type 2 (HER2) are currently the most frequently used targets in NIR-PIT 28-32. The ability of NIR-PITs to eliminate EGFR- or HER2-expressing cancer cells have been used to treat multiple types of tumors, including brain 33, head and neck 34, 35, bladder 36-38, lung 39-42, ovarian 43-45, breast 46-50, esophageal 51, and gastrointestinal 52-56 cancers. Tumor-associated antigens (TAAs), such as carcinoembryonic antigen (CEA) for gastric, pancreatic, and colorectal 12, 57, 58 cancers, glypican-3 (GPC3) for hepatocellular carcinoma 59, 60, prostate-specific membrane antigen (PSMA) for prostate cancer 61-64, CD20 for B-cell lymphoma 65, 66 and CD146 for melanoma 67, are also attractive targets for NIR-PIT. Indeed, the efficacy of targeting some of these antigens has been assessed clinically for tumor imaging and treatment. Drug-resistance-related proteins are another important type of antigen that can be used for NIR-PIT. For example, P-glycoprotein (Pgp) mediates multidrug resistance in numerous cancers by pumping anticancer drugs out of cancer cells 68. As such, Pgp is an attractive target for NIR-PIT due to its critical function and selective expression by cancer cells. In xenograft models of chemoresistant cancer, anti-Pgp antibodies and anti-Pgp Fab-mediated NIR-PIT elicit comparable spatially selective tumor ablation, and prolong the survival of treated mice 69, 70. By contrast, Fab conjugates require a shorter time to obtain peak accumulation and are distributed more homogeneously in tumors, which is beneficial for clinical translation 70. When combined with liposomal doxorubicin (Doxil), an FDA-approved cancer nanodrug, Pgp-targeted NIR-PIT improves the therapeutic effect of Doxil by depleting multidrug resistant (MDR) cancer cells and increasing the tumor penetration of Doxil 71. Surface markers of cancer stem cells (CSCs), such as CD44 72, 73 or CD133 74, have been used in NIR-PIT to kill aggressive subpopulations of cells in the tumor, resulting in good therapeutic effects. The expression of immune evasion-related CD47 on tumor cells can serve as a “don't eat me” signal after binding to its receptor SIRPα expressed on phagocytes. Interestingly, anti-CD47 antibody-directed NIR-PIT was found to not only cause phototoxicity but also induce phagocytosis by blocking the CD47-SIRPa interaction 75.

### Therapeutic targets on non-tumor cells in the tumor microenvironment (TME)

NIR-PIT can be designed to damage components of the TME, including vascular cells, cancer-associated fibroblasts (CAFs), and tumor-infiltrating immune cells, such as regulatory T cells (Tregs) and unwanted phagocytes 75-77. NIR-PIT targeting tumor blood vessels can mediate tumor cell death by blocking the supply of nutrients and oxygen 56, 78-81. However, severe damage to tumor blood vessels induced by NIR-PIT might also hinder further drug delivery into the tumor parenchyma. Nevertheless, whether NIR-PIT-induced neovasculature damage inhibits immune activation, and the SUPR effects, remain to be investigated.

Unlike tumor/vasculature-specific antibody-mediated NIR-PIT, CD25-targeted NIR-PIT has been used to selectively eradicate tumor-infiltrating Tregs—a type of immunosuppressive cell with high CD25 expression—to treat a broad range of cancers in murine models, including lung, colon, and prostate cancers 82-84. The antitumor effect of CD25-targeted NIR-PIT involves activation of effector CD8^+^ T cells and NK cells, which are present in the TME but suppressed by Tregs. Hence, by selective depletion of Tregs, CD25-targeted NIR-PIT not only induces regression of irradiated tumors, but also elicits a therapeutic response against distant non-treated tumors in a tumor-antigen-specific manner. Additionally, repeated applications of NIR-PIT are feasible, which may not be the case for ionizing radiation. Despite CD25 upregulation in activated CD8^+^ T and NK cells, CD25 expression on these effector cells is downregulated after repeated NIR-PIT following a 4-day interval 84. However, it is worth noting that CD25-targeted NIR-PIT could be less effective in cancers where Tregs do not play a major role. Meanwhile, CD73 (extracellular-5′-nucleotidase) is overexpressed in many cancer cells as well as TME-related cells, such as Tregs, myeloid-derived suppressor cells (MDSCs), and tumor-associated macrophages, however, is absent in effector CD8^+^ T cells, NK cells and DCs. Thus, by locally depleting multiple immunosuppressor cell types and significantly increasing CD8^+^ effector T cells in tumors, the CD73-targeted NIR-PIT + anti-PD1 antibody combination treatment could overcome acquired resistance to PD-1 blockade, and eradicate advanced tumor xenografts in mice, irrespective of CD73 expression levels in tumor cells 77. Another immunotherapy-related target molecule is V-domain immunoglobulin suppressor of T cell activation (VISTA), which is an immune checkpoint molecule of the B7 ligand family. VISTA is broadly expressed on Tregs and MDSCs of tumor-infiltrating lymphocytes, as well as various primary human tumors. VISTA-targeted NIR-PIT inhibits tumor growth and prolongs survival in murine tumor models by activating CTLs and DCs in the TME. Taken together, by editing the tumor microenvironment, NIR-PIT can dramatically activate systemic antitumor immunity against both the treated and the untreated distant tumors 85.

### Combined targeting of multiple targets or epitopes

TSPCs recognizing different antigens can be combined in NIR-PIT treatment to enhance the therapeutic effects 49, 53, 86. Due to tumor heterogeneity, NIR-PIT against a single target may not eradicate all tumor cells and may not consistently achieve therapeutic effects in different patients. Thus, NIR-PIT against multiple molecular targets, such as the combination of EGFR with HER2 for bladder cancer therapy 86, as well as a cocktail of EGFR, epithelial cell adhesion molecule (EpCAM), and chondroitin sulfate proteoglycan 4 (CSPG4) for the treatment of triple-negative breast cancer (TNBC) 49, exhibit therapeutic effects superior to NIR-PIT against a single target. Moreover, antigens on different cell populations in the TME can be targeted simultaneously in NIR-PIT to improve therapeutic efficacy. NIR-PIT exploiting anti-CD44 and anti-CD25 antibodies significantly inhibits tumor growth and prolongs survival in several tumor animal models compared with NIR-PIT based on a single target, owing to the depletion of both cancer cells and immunosuppressive cells in the TME 83. A combination of nanobody-mediated NIR-PIT, which targets vascular endothelial growth factor receptor 2 (VEGFR2) on tumor endothelial cells, and EGFR on tumor cells, has also shown improved efficacy in a coculture model of endothelial and cancer cells 87. In addition to targeting different antigens, NIR-PIT can act on different epitopes of the same antigen. For instance, the HER2-targeted antibodies trastuzumab and pertuzumab increased the specific delivery of IR700 to HER2-positive tumor cells with no competition for antigen binding, thus leading to a stronger anti-tumor response after NIR exposure 53. Similarly, a bispecific nanobody designed by engineering monovalent nanobodies into a biparatopic construct could recognize two non-overlapping epitopes on the HER2 molecule. NIR-PIT using this nanobody not only enhances therapeutic effects compared with NIR-PIT targeting individual epitopes, but also eradicates trastuzumab-resistant HER2-positive breast cancer, providing a promising new approach for future clinical practice 88. Therefore, it is likely that multiple TSPCs targeting different antigens or epitopes will achieve an augmented antitumor effect.

## Construction of target-specific PSs

TSPCs with different sizes, affinities, and pharmacokinetics can influence the therapeutic effects of NIR-PIT. An intact antibody has a relatively large size, and thus exhibits poor penetration in solid tumors, limiting the therapeutic efficacy. In this regard, genetically engineered small antibody fragments may retain a high affinity for the antigen while improving the intratumoral delivery of TSPCs. Moreover, the smaller size can enable rapid accumulation of TSPC in the tumor along with rapid systemic clearance. Generation of TSPCs comprising with a diabody (Db), minibody (Mb) 89, Fab 70, 82, 90, nanobody 35, 91, 92, affibody 78, 93, peptide 79, 80, small molecule 63, 94, or recombinant virus-like particles (VLPs) 95, exhibit antitumor effects similar to, or better than, those shown by NIR-PIT with a full-size IgG antibody. However, TSPCs based on whole antibodies are also likely to exhibit a higher affinity for antigens and superior tumor retention compared with their fragments, and could be more suitable for repeated light exposure to optimize therapeutic efficacy without increasing side effects 62.

Shirasu et al. reported an interesting universal TSPC construction, expanding the applicability of NIR-PIT. They conjugated IR700 with NeutrAvidin, which specifically binds to a range of biotinylated tumor-homing molecules. This TSPC indirectly binds to targeted cells with the help of anti-CEA or anti-EpCAM biotinylated antibodies and exhibits cytotoxicity levels, after light irradiation, similar to those elicited by other NIR-PITs, which TSPCs bind directly to the cell membrane 96.

As discussed previously, the affinity of TSPC can affect the antitumor response of NIR-PIT due to the “binding site barrier” 26. Hence, when constructing TSPCs, it is important to consider the balance between the affinity and tumor penetration to enhance therapeutic efficacy. For example, in a study on nanobody-based NIR-PIT, the researchers altered the affinity of their TSPCs by varying the number of arginine-glycine-aspartic acid (RGD) peptides that could specifically bind to integrins on the cell membrane. They revealed that a polymer containing cyclic 15-tandem repeats of RGD peptides in one construct permitted preferential accumulation in the tumor without compromising penetration to deeper areas, when compared with 5-repeat RGD peptides 80.

The pharmacokinetics of TSPC also affect NIR-PIT efficacy. A previous study compared TSPCs constructed using different anti-EGFR antibodies. Those TSPCs with two antibodies, namely, cetuximab (cet) and panitumumab (Pan), exhibited equal affinity and identical phototoxicity *in vitro*. However, Pan-IR700 exhibited an antitumor response superior to that of cet-IR700 *in vivo*. Examination of the biodistribution revealed that cet-IR700 had lower tumor accumulation and more rapid hepatic catabolism than Pan-IR700 97. Hence, they concluded that both agents could be useful in certain situations. For instance, the shorter half-life of cet-IR700 might lead to fewer safety concerns, while the longer retention of Pan-IR700 in the circulation could achieve better penetration into the tumor when repeated NIR-PIT is applied.

Notably, when TSPCs specifically bind to their targeted receptors, they may transiently block receptor function, thereby, altering therapeutic efficacy. In a study that eradicated Tregs using anti-CD25-targeted NIR-PIT, an IgG-directed TSPC was reported as less effective than an F(ab')_2_-guided TSPC. F(ab')_2_-mediated NIR-PIT recruits a higher number of infiltrating lymphocytes to the tumor. The slower clearance of anti-CD25-IgG-IR700 might account for its lower efficacy, as the sustained circulation of anti-CD25-IgG-IR700 can impair the binding of activated effector T cells to IL-2, a cytokine that promotes T cell proliferation 82. Other factors, such as solvent pH and conjugation ratio, can also impact the stability and efficacy of TSPCs 98.

## NIR light irradiation

NIR light is an important component of NIR-PIT. While the light dose in NIR-PIT is typically between 50 and 150 J/cm^2^ 18, 50, 99, increasing the light dose leads to more potent cytotoxicity. However, the power density (mW/cm^2^) of the light source is not as critical to the effectiveness of NIR-PIT as the total light dose (J/cm^2^) 100. When comparing light sources, LEDs have proven less effective than lasers 101.

Researchers are currently seeking improved strategies to deliver NIR light. In addition to delivering NIR irradiation from outside the body, endoscopic delivery is also being explored 54, 55. In particular, this approach could achieve better efficacy against tumor growth and its dissemination to the lungs 39 or body cavities, such as the bladder, abdominal cavity, or pleural cavity. Meanwhile, applying NIR-PIT during surgery allows for direct exposure of the tumor bed to NIR light as well as removal of any residual tumor. Implantable wireless-powered LEDs have also been developed to treat tumors in deep tissues. However, to address safety concerns, the power supply is separated from the light system. In fact, a newly developed wireless light system can emit NIR light up to 20 cm away from the power supply, thus covering most lesions in the human body. Moreover, this LED system can irradiate tumors over an extended period of time 99.

## Combinational strategies for enhancing the antitumor effect of NIR-PIT

A commonly used strategy to improve the antitumor effect is combining NIR-PIT with other therapeutic agents, such as nanodrugs 80, chemotherapeutic drugs 52, NIR light-responsive drugs (e.g., CyEt-panitumab-duocarmycin) 47, and immune checkpoint inhibitors 90, 102. The SUPR effects or the synergistic antitumor mechanisms may enhance the antitumor effects of these combinations. CD276 is a transmembrane glycoprotein overexpressed in both tumor cells and the tumor vasculature. Interestingly, CD276-mediated NIR-PIT increases the activation and maturation of DCs, and markedly increases the level of programmed cell death protein 1 (PD-L1) in tumors. When combined with αPD-L1 treatment, NIR-PIT enhances therapeutic efficacy by generating local and systemic antitumor immunity 90. Maruoka et al. combined CD44-targeted NIR-PIT with a PD-1/PD-L1 axis blockade 102, cytotoxic T lymphocyte-associated antigen-4 (CTLA4) blockade 103, or IL-15 treatment 104. These three combinations showed enhanced antitumor effects compared with each therapy alone. Notably, CD44-targeted NIR-PIT combined with CTLA4 or PD-1/PD-L1 blockade showed varying antitumor responses in different tumor models. For instance, in MOC1 tumors, 44% complete remission (CR) was achieved with NIR-PIT and CTLA4 blockade, compared with only 8% when using NIR-PIT and PD-1 blockade. However, in MC38-luc tumors, a CR of only 11% was achieved with NIR-PIT and CTLA4 blockade, while a CR of 70% occurred with NIR-PIT and PD-1 blockade 103. Given the heterogeneity of immune cell populations in different tumors, the most appropriate immune checkpoint inhibitors to be combined with NIR-PIT could be selected based on the expression level of specific immune checkpoint molecules in the respective tumors. For instance, Hartmans et al. combined tyrosine kinase inhibitors (TKIs) and EGFR/HER2-targeted NIR-PIT and found that TKIs upregulate EGFR expression, which contributes to the improved antitumor effect 51. In addition to the combinational strategy, cytotoxic drugs can be directly incorporated into TSPCs by constructing antibody-drug conjugates (ADCs). Trastuzumab emtansine (T-DM1) is an ADC comprising the anti-HER2 monoclonal antibody trastuzumab conjugated with the cytotoxic drug maytansinoid DM1 48. In this way, a T-DM1-IR700 conjugate with two different cell-killing payloads was developed that enhances cytotoxicity *in vitro* and antitumor effects against large tumor xenografts (~200 mm^3^) *in vivo*.

## Potential application of NIR-PIT for cancer treatment

**Figure 4:**
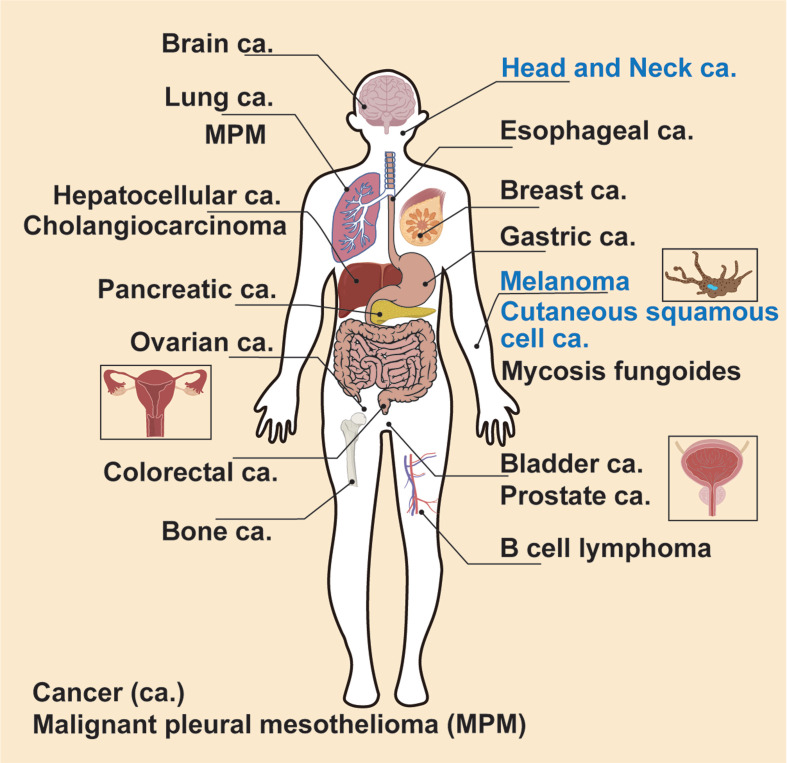
An overview of NIR-PIT for treating 19 different types of cancers, including the clinical applications or ongoing clinical trials for treatment of head and neck squamous cell carcinoma, melanoma, and cutaneous squamous cell carcinoma.

In this section, we review the application of NIR-PIT in cancer treatment (Figure 4 and Tables 1-3). The targets, efficacy, and NIR-PIT approach are described for each cancer type. To assist in locating each cancer type, they have been arranged alphabetically.

### B-cell lymphoma

CD20 is a B-cell specific antigen highly expressed in many B cell lymphomas (BLs) and was, therefore, selected as an important target for treating BL 105. The lack of shedding or internalization makes CD20 an excellent candidate for NIR-PIT. Anti-CD20 NIR-PIT has shown efficacy in both indolent and aggressive BLs. *In vitro* studies suggest that aggressive BL is more sensitive to NIR-PIT than indolent BL. In animal models using two human aggressive BL cell lines (Daudi and Ramos), NIR-PIT significantly inhibits tumor growth and prolongs survival compared with control groups 65. For aggressive BL, NIR-PIT elicits a strong therapeutic response than radioimmunotherapy, which has been proven effective in mice bearing Ramos tumor grafts. 66, 106. However, NIR-PIT is difficult to implement for treatment of other hematological malignancies without formation of a local mass.

### Bladder cancer

EGFR is highly expressed in 74% of bladder cancer tissue specimens 28. NIR-PIT using the anti-EGFR antibody panitumumab led to inhibition of tumor growth in a mouse model and completely shrank the tumor size in three out of ten xenograft mice. Moreover, the outcome of NIR-PIT correlates with the level of EGFR expression on tumor cells 36.

Researchers have also made efforts to develop a larger animal model for translational studies and to investigate cystoscopy for NIR light delivery. As spontaneous bladder cancer in dogs closely mimics human invasive bladder cancer 107, a canine version of the anti-EGFR antibody cetuximab, can225IgG linked to IR700 (can225-IR700), was tested in a canine tumor-bearing xenograft mouse model. A single injection of can225-IR700 with two rounds of light exposure led to remarkable therapeutic response 38.

HER2 is a member of the tyrosine kinase EGFR family that promotes cell proliferation, differentiation, and motility. It is commonly overexpressed on various types of cancer cells, and has been identified in 40% of bladder cancer specimens 108. Moreover, a cocktail of NIR-PIT performed significantly better targeting EGFR and HER2 together, than when targeting either alone, both *in vitro* and *in vivo*. However, 4/11 mice died within 48 h following irradiation with the higher dose light (100 J/cm^2^) in the combination group. Indeed, excessive light exposure or TSPC dosage beyond the maximum tolerated doses (MTDs) can result in death of mice. Thus, the potential treatment toxicity with drug dose-escalation must be considered to achieve the optimal antitumor response. Subsequent toxicity assays indicate that tumor lysis syndrome (TLS), caused by massive tumor necrosis, provoked a strong systemic inflammatory response in the mice that died 86. However, in clinical trials, no incidence of TLS was observed in cancer patients treated with NIR-PIT 109, 110. Moreover, aggressive intravenous hydration and administration of hypouricemic agents significantly decrease the incidence of poor outcomes with TLS 111. Nevertheless, a balance must be achieved between optimum antitumor effects and minimal toxicity to healthy tissues prior to clinical application.

CD47—a ligand that transmits inhibitory signals for macrophage phagocytosis—is overexpressed on bladder cancer cells, however, is absent in the normal luminal urothelium 112. Anti-CD47 NIR-PIT causes cell necrosis in patient-derived bladder cancer cells and human bladder cancer cell lines, and significantly inhibits tumor growth in a xenograft model 75. Furthermore, NIR-PIT does not compromise anti-CD47 antibody binding and has the potential to enhance phagocytosis by increasing the surface expression of calreticulin, which can counterbalance the inhibitory signal of CD47 75, 113.

### Bone cancer and bone metastasis

Nakamura et al. chose an explanted bovine rib as a model to determine whether NIR-PIT can effectively kill cancer cells within bones. Their *ex vivo* experiments showed that although the transmittance of NIR light was only 4.52%, this therapy could potently destroy cancer cells behind the rib 114. Meanwhile, given that human bone samples have been reported to have a markedly lower density than bovine ribs, NIR-PIT should theoretically perform better in human bone cancer 115.

### Brain cancer

Considering that EGFR is overexpressed in approximately 50% of primary glioblastomas (GBMs), it represents an interesting target for GBM therapy 29. Burley et al. developed a low-molecular-weight (~7 kDa) EGFR-specific affibody to be conjugated with IR700 (Z_EGFR:03115_-IR700DX). This conjugate showed high specificity for binding to EGFR and significant therapeutic efficacy after light irradiation, both *in vitro* and in a xenograft model of GBM 33.

G protein-coupled receptors (GPCRs), including US28 and type 2 cannabinoid receptor (CB2R), are abnormally overexpressed in multiple tumors, including brain tumors 116, 117. Meanwhile, a nanobody specifically binding to the extracellular region of US28 was developed to be conjugated with IR700, which potently mediated NIR-PIT killing of US28-expressing glioblastoma cells both in 2D and 3D cultures after NIR exposure 92. NIR-PIT treatment with mbc94, a CB2R-specific molecule, was efficient and target-selective in delaying the growth of brain tumor cells both *in vitro* and in a xenograft model 118, 119.

CSCs are undifferentiated tumor cells with high self-renewal capacity, limited differentiation capability, and high motility 120, 121. CD133 is a CSC marker in many tumor types, including GBM 122. Hence, NIR-PIT with anti-CD133-IR700 was tested in a mouse xenograft model using orthotopic gliomas initiated from patient-derived CSCs. The treatment was highly effective and prolonged survival in the orthotopic model, although cerebral edema was observed several hours after NIR-PIT, which may have been due to tumor necrosis 74.

### Breast cancer

HER2 is highly expressed in approximately 20% of human breast cancers and is generally associated with poor outcomes 123. Thus, HER2-targeted NIR-PIT may prove to be a promising option for breast cancer treatment. Compared with the intact antibody, the small affibody (6-7 kDa) exhibits superior tumor penetration and rapid clearance. Meanwhile, the HER2 affibody-IR700 conjugate leads to necrotic cell death only in HER2-overexpression cancer cells following NIR light irradiation, without causing collateral damage to HER2 low-expressing cancer cells 93, 124. In addition, combination of the HER2 affibody-based conjugate and the trastuzumab-IR700 conjugate induces a strong therapeutic effect against HER2-positive cancer cells, including HER2 low-expressing cells, trastuzumab-resistant cells, and brain metastatic breast cancer cells 125.

Anti-EGFR-based NIR-PIT has also been investigated for the treatment of breast cancer. In an orthotopic breast tumor model, panitumumab conjugated with IR700 for NIR-PIT immediately induced the killing of >90% cells *in vivo* after irradiation at a high light dose (>100 J/cm^2^) 50. Nani et al. used an NIR photocaged-group-based ADC, CyEt-panitumumab-duocarmycin, to selectively trigger the release of the cytotoxic drug duocarmycin after NIR-PIT to enhance tumor-specific therapeutic effects 126. In this study, NIR-triggered drug release was achieved after panitumab-mediated NIR-PIT, and the SUPR effects contributed to the increased ADC tumor accumulation and high tumor-background ratio 47.

TNBC is defined by the lack of expression of estrogen receptor (ER), progesterone receptor (PR), and HER2, and is associated with shorter relapse-free intervals and lower overall survival than other subtypes. Moreover, no targeted therapy is currently available for TNBC, in contrast to ER-positive or HER2-positive breast tumors. Fortunately, 50-70% of TNBCs express EGFR 127 and the anti-EGFR antibody cetuximab (cet)-IR700-based NIR-PIT has demonstrated the ability to suppress tumor growth and significantly prolong patient survival regardless of the EGFR expression level in the tumors, although higher expression of EGFR induces increased cell death. Moreover, the therapeutic effect can be further enhanced by splitting the TSPC dose and repeating the NIR exposure 46.

A cocktail of NIR-PIT targeting three different receptors has also been investigated for the treatment of TNBC. These receptors, including EGFR, EpCAM, and chondroitin sulfate proteoglycan 4 (CSPG4), are abundantly expressed in TNBC, but only at low levels in normal tissues 128-130. The combination of all three scFv-IR700 conjugates increases phototoxicity by up to 40% compared with each photoimmunotherapy agent *in vitro* 49.

The high recurrence and metastasis rates of TNBC are due in part to the presence of CSCs, which are resistant to conventional therapy 129. Moreover, CD44 is a CSC marker in many cancer types, including breast cancer 131, and its overexpression results in a more aggressive phenotype 132. Hence, the conjugation of anti-CD44 antibody and IR700 induces marked tumor shrinkage and significant cell death in CD44-positive breast cancer cell populations following light exposure 72. However, hypoxia can upregulate CD44 expression 133 and induce cancer cell resistance to antitumor therapies, such as radiation and chemotherapy. Nevertheless, hypoxia does not compromise NIR-PIT efficacy, which destroys the cell membrane without the need for oxygen 72. Indeed, NIR-PIT using T-DM1-IR700 (an ADC-based TSPC) exhibits superior antitumor effects in HER2-positive large tumor models even when NIR light could not sufficiently irradiate the whole large tumor 48.

### Cholangiocarcinoma (CCA)

Type I transmembrane protein tumor-associated calcium signal transducer 2 (TROP2) has been tested as a therapeutic target in pancreatic carcinoma (PC) and CCA. NIR-PIT with an anti‐TROP2 antibody significantly inhibits PC and CC xenograft tumor growth. In the same study, TROP2 was identified in 40% of PC samples and 46% of CCA samples, indicating that half of the patients could be covered 134. However, light delivery may be hindered in CCA due to its location within the body. Accordingly, Hirata et al. developed an NIR-LED catheter intended for use within the bile duct, equipped with a temperature sensor to prevent thermal burns. NIR-PIT, using the novel catheter device inserted under subcutaneous tumor xenografts in mice, exhibits therapeutic effects comparable to those of the external irradiation device. However, this device has not been assessed in the murine bile duct for CCA treatment due to the small size of murine bile ducts 135.

### Colorectal cancer (CRC)

Elekonawo et al. evaluated the effects of CEA-targeted NIR-PIT in nine CRC cell lines expressing different levels of CEA. Their results indicate that the antitumor efficacy of NIR-PIT is proportional to CEA expression level 136. Another group investigated CEA-targeted NIR-PIT in an orthotopic colon cancer mouse model and observed clear inhibition of tumor growth 58.

Moreover, glycoprotein A33 antigen (GPA33), which is overexpressed in human CRC, has also been investigated as a target for NIR-PIT 137. The single-chain variable fragment of an antibody against GPA33 (A33scFv) conjugated to IR700 (A33scFv-IR700**)** exhibits rapid and specific killing of GPA33-positive colorectal tumor cells after NIR laser light irradiation *in vitro*. In mice bearing GPA33-positive tumor xenografts, A33scFv-IR700-mediated NIR-PIT exhibits significant antitumor effects after a single NIR exposure 138. Platelet-derived growth factor receptor β (PDGFRβ) can be used in NIR-PIT for colon cancer as it is abundantly expressed on the pericytes of various types of tumors 139. Although PDGFRβ is also expressed in normal tissues, it is safe to use as a target as endothelial cells completely cover the pericytes in normal blood vessels. Meanwhile, endothelial cells irregularly cover the vascular wall only in tumor blood vessels, permitting the delivery of PS to exposed pericytes 140. Moreover, PDGFRβ-specific dimeric affibody is cheaper and smaller than a full length-antibody. NIR-PIT using this affibody successfully damages tumor blood vessels, leading to thrombosis or even vessel occlusion. In addition, the resulting intensified tissue hypoxia helps destroy the tumor 78.

Since EGFR is also highly expressed in most CRC cells, an EGFR-targeting affibody was utilized as an NIR-PIT agent combined with long-acting tumor necrosis factor-related apoptosis-inducing ligand (TRAIL) to treat chemotherapy MDR and TRAIL resistance in CRC. In this study, EGFR-targeted NIR-PIT significantly increased the sensitivity of MDR cells to TRAIL, and the combination therapy efficiently eradicated large CRC xenografts (~150 mm^3^) with both chemotherapy MDR and TRAIL resistance 141.

### Cutaneous squamous cell carcinoma (CSCC)

NIR-PIT is suitable for the treatment of skin disorders as the light can be directly irradiated. In the case of CSCC, EGFR 19, 20, 27, 45, 97 and mesothelin 142 have been used as targets for TSPCs, which specifically recognize and kill A431 cells after exposure to NIR light, both *in vitro* and in xenograft-bearing mice. Moreover, an open-label clinical trial administering cetuximab-mediated NIR-PIT combined with PD1 blockade to patients with advanced or metastatic CSCC is currently underway (NCT04305795).

### Esophageal cancer (EC)

Growth factor receptors, including EGFR and HER2, can act as targets of NIR-PIT in EC. TSPCs targeting these growth receptors showed good phototoxicity *in vitro*. Researchers have also found that the therapeutic effects of NIR-PIT can be further enhanced using a TKI to upregulate the expression of growth factor receptors 51. Many studies have reported that CAFs are associated with therapeutic resistance in EC. In fact, CAFs have been identified as a crucial component of the TME that contributes to tumor progression 143, 144. Meanwhile, anti-FAP NIR-PIT showed safe and promising inhibitory effects on fibroblast activation protein (FAP, a specific marker of CAFs 145)-positive CAFs *in vitro* and *in vivo* 76. Another study showed that the coculture of EC cells and CAFs results in a CAF-driven resistance to chemotherapy. Moreover, a combination of 5-FU with CAF-targeted NIR-PIT can result in a 70.9% drug-resistant tumor shrinkage, whereas treatment with 5-fluorouracil (5-FU) alone only elicits 13.3% tumor shrinkage 146. These results suggest that NIR-PIT-targeting CAFs has the potential to overcome resistance to conventional chemotherapy.

### Gastric cancer

Several studies have tested NIR-PIT to treat peritoneal dissemination of gastric cancer because NIR light can be distributed to multiple cancer sites simultaneously in the peritoneal cavity via endoscopy. Given that approximately 20% of gastric cancers express HER2, these studies utilized anti-HER2 NIR-PIT 30, 31. NIR-PIT using a fiber optic diffuser showed good therapeutic effects in a mouse model of disseminated peritoneal gastric cancer. Meanwhile, histological analysis revealed diffuse cancer cell death in treated tumor tissues, and prolonged survival following repeated NIR-PIT 54, 55. This strategy might be easier to execute in human patients than mouse models due to the greater flexibility associated with performing endoscopies in the larger peritoneal space of humans. Moreover, compared with NIR-PIT alone, the addition of 5-FU to HER2-targeted NIR-PIT elicits significant cytotoxicity in both short-term and long-term follow up. The enhanced and prolonged tumor growth inhibition *in vivo* can be attributed to the difference in cell killing mechanisms, namely, necrotic membrane damage and apoptotic cell death 52.

Gastrointestinal stromal tumors (GISTs) are the most common type of submucosal tumors of the gastrointestinal tract, with up to 90% of GISTs overexpressing c-KIT (a receptor tyrosine kinase also known as CD117) 147. Thus, anti-c-KIT-based NIR-PIT has been used for the imaging and treatment of GISTs. Tumor-specific accumulation of IR700-conjugated anti-c-KIT antibodies has been observed in mice bearing subcutaneous GIST xenografts, while GISTs have been visualized using fluorophore AF48-conjugated anti-c-KIT antibody through the normal mucosa of an orthotopic rat model. Additionally, effective cell killing has been reported *in vitro* and in mouse models 148. Thus, the IR700-conjugated anti-c-KIT antibody could be a promising theranostic agent for GISTs.

VEGFR-2 expressed on the vascular endothelium has also been evaluated as a potential target of NIR-PIT for gastric cancer. In fact, anti-VEGFR2-mediated NIR-PIT induces damage in the tumor neovasculature of xenograft tumors. Compared with anti-HER2 NIR-PIT, VEGFR2-targeted NIR-PIT has a stronger antitumor effect, although this approach leads to a lower amount of TSPC inside the tumor. However, vessel destruction may fail to induce SUPR effects and the subsequent immune response achieved with anti-HER2 NIR-PIT. Moreover, although VEGFR2 is also expressed in normal tissues, NIR light can be applied locally, thus limiting normal tissue toxicity 56.

### Head and neck cancer

Due in part to the complex topography of the regional anatomy and the abundance of vital structures, the recurrence rate of squamous cell carcinoma of the head and neck (SCCHN) remains high even after administration of the gold standard treatment, i.e., surgical resection plus adjuvant radiotherapy, or chemoradiation 149. Meanwhile, EGFR-targeted NIR-PIT has the potential to eliminate microscopic tumor remnants in a postsurgical setting. In a study performed by Moore et al., NIR-PIT using pan-IR700 was investigated *in vitro,* and in an *in vivo* model of residual disease after incomplete resection. Mice that received surgical treatment plus adjuvant NIR-PIT showed a significant (*P* < 0.001) reduction in tumor regrowth 30 days post-NIR-PIT in both the 50% and 90% subtotal resection groups. However, tumor recurrence ultimately occurred in all treated mice. Hence, a more potent NIR-PIT regimen, such as repeated light exposure, may be needed 34. In addition to the full antibody, 7D12, a small nanobody targeting EGFR has been used in NIR-PIT for head and neck cancer. The 7D12 nanobody has a shorter half-life and deeper tumor penetration than the intact antibody. 7D12-mediated NIR-PIT resulted in significant tumor necrosis and infiltration of immune cells within 1 h of NIR-PIT, however, showed no toxicity to normal tissues 35. CD44 is a CSC marker that is selected for treatment of oral cavity squamous cell carcinoma. A single injection of the CD44-targeted TSPCs followed by light exposure induced a potent antitumor effect *in vivo* 73.

### Hepatocellular carcinoma (HCC)

Glypican-3 (GPC3) is an attractive antigen for treating HCC as its expression in HCC is higher than that in normal liver 150. Hanaoka *et al.* compared NIR-PIT using the whole IgG antibody anti-GPC3 YP7 with that using the human heavy-chain antibody anti-GPC3 HN3 and found that both groups showed significant inhibition of tumor growth *in vivo*. However, the HN3-IR700 conjugate provided a deeper, and more homogeneous, distribution in tumors thanYP7-IR700, without compromising the affinity of HN3 for GPC3. Moreover, HN3-IR700 retained its capacity to induce antibody-dependent cell-mediated cytotoxicity (ADCC). Hence, researchers may prefer to use HN3 rather than YP7 in NIR-PIT for HCC treatment. Considering that light delivery can be problematic in HCC, light probes placed through vessels or inserted directly through the skin into the liver (such as conventional interventional angiography or focal laser ablation) could be necessary for effective NIR-PIT 59. Additionally, YP7-mediated NIR-PIT in combination with albumin nanoparticle-bound paclitaxel has been shown to increase the antitumor efficacy via SUPR effects 60.

### Lung cancer

The lungs are considered suitable for light therapy due to the relative ease of light penetration into deep air-pockets without significant light attenuation. In a transgenic model of spontaneous lung cancer, tumor growth was significantly inhibited in the anti-EGFR NIR-PIT group compared with the control group (*P* < 0.01 at all time points) 40. In another mouse model of pleural disseminated non-small-cell lung carcinoma, anti-HER2 NIR-PIT led to a significant reduction in tumor volume (*P* = 0.002 vs. TSPC) and prolonged survival (*P* < 0.0001) 42. Delta-like protein 3 (DLL3) was also selected as a potential target for small cell lung cancer (SCLC). Moreover, anti-DLL3 NIR-PIT led to extensive cell death *in vitro* and a remarkable decrease in tumor volume in a xenograft model 151. PD-L1 is highly expressed in many cancers, leading to suppression of T-cell activation and unimpeded tumor growth 152. In addition, PD-L1 overexpression is associated with poor prognosis 153. Hence, the anti-PD-L1 antibody has also been used as an NIR-PIT carrier. PD-L1-targeted NIR-PIT induces significant inhibition of tumor growth and prolonged survival in a PD-L1-expressing lung papillary adenocarcinoma model, even with a single treatment 154.

Considering that the lungs are among the most common sites for developing metastases, NIR-PIT has also been used to treat lung metastases. In fact, in a murine model, anti-HER2 trastuzumab-based NIR-PIT demonstrated a significant reduction in early-stage lung metastatic disease (*P* < 0.0001) without damaging non-targeted tumors or normal lung tissues 41. Moreover, a significant reduction in tumor size and prolonged survival was achieved using NIR-PIT in a mouse model with large lung metastases of ~100 mm^3^ in volume 39. When translated into humans, bronchoscopy or direct thoracoscopy may be required to deliver NIR light to reach the entire lung volume. Moreover, as pulmonary metastasis is a systemic disease, NIR-PIT could be applied as a palliative treatment to relieve symptoms, which may lead to longer survival and a better quality of life.

### Malignant pleural mesothelioma (MPM)

The poor prognosis and few available therapeutic approaches for MPM have attracted interest in the possible application of NIR-PIT in MPM. Podoplanin (PDPN), a type I transmembrane glycoprotein, is used as a specific pathological diagnostic marker for MPM. Anti-PDPN antibody NZ-1-mediated NIR-PIT induces light-dose-dependent cytotoxicity* in vitro* and good therapeutic effects *in vivo*, in both flank and orthotopic xenograft models 155. However, NZ-1 also binds to normal lymphatic endothelial cells and can potentially harm normal cells. Hence, a novel mAb (CasMab) has been developed to distinguish cancer cells from normal cells by recognizing differential expression of glycosylated PDPN 156. Thus, NIR-PIT employing CasMab may achieve a superior antitumor effect in the treatment of MPM.

### Melanoma

Melanoma is one of the most fatal types of skin cancer. Given that CD146 is upregulated in melanomas and is associated with progression and metastasis 157, anti-CD146 NIR-PIT has been applied to xenograft melanoma tumors of varying sizes. Compared with the control group, after treatment with anti-CD146 NIR-PIT, significant suppression of tumor growth was observed in small xenografts (86.48 ± 14.80 mm^3^), whereas no significant difference was detected in large xenografts (330.80 ± 36.12 mm^3^) 67. The poor therapeutic response elicited by NIR-PIT for the treatment of large melanomas may be due to the finite TSPC delivery and limited NIR light penetration into tumors 158. Irradiation of large tumors can be performed using optical fibers inserted through needle catheters, which have been applied to treat head and neck squamous cell carcinoma (HNSCC) patients in clinical trials 109. Moreover, repeated NIR exposure can improve the delivery of TSPC into the tumor bed via SUPR effects 24. Additionally, combinatorial therapies comprising NIR-PIT and conventional immunotherapy may achieve a synergistic suppressive effect on tumor growth for melanomas of larger volumes 8.

Furusawa et al. investigated the efficacy of CD29-targeted NIR-PIT in the highly pigmented melanoma model. CD29 (also named integrin beta-1, ITGβ1) is a cell surface glycoprotein that is expressed on both melanoma and cytotoxic T cells. CD29-targeted NIR-PIT efficiently suppresses tumor growth in the pigmented melanoma model. More importantly, NIR-PIT does not cause damage to immune cells at the treatment site while preferentially killing proliferative cancer cells. Moreover, the combination of an anti-CTLA4 antibody and CD29-targeted NIR-PIT increases the infiltration of CD8^+^ T cells, resulting in greater tumor growth inhibition with prolonged survival 159.

Virus-like particles (VLPs) and IR700 have been employed to construct a TSPC (AU-011) for the treatment of primary uveal melanoma, leading to significant dose-dependent tumor necrosis after NIR irradiation. According to Kines et al., VLPs were prepared based on the HPV capsid protein. VLP attachment and internalization by targeted cells is mediated by heparan sulfate proteoglycan (HSPG) expressed on multiple tumor cell types. VLPs have two advantages over antibodies for NIR-PIT treatment. First, they exhibit multivalent binding, which enables stronger interaction with target cells. Second, a large number of IR700 dye molecules can be readily conjugated to a single VLP, leading to a higher concentration of PS on the cell surface 95.

### Mycosis fungoides (MF)

MF is a low-grade T-cell lymphoma, accounting for approximately 53% of primary cutaneous lymphomas 160. Few therapies are available for MF treatment; therefore, new therapeutic approaches are necessary. A study by Silic-Benussi et al. investigated anti-cutaneous lymphocyte antigen (CLA)-targeted NIR-PIT, which effectively destroyed MF-derived My-La CD4^+^ cells *in vitro* 161.

### Ovarian cancer

Patients with intraperitoneal tumor dissemination of ovarian carcinoma, commonly present at an advanced stage and have a poor prognosis 162. However, the antitumor effect of anti-HER2 NIR-PIT was investigated in a mouse model of disseminated peritoneal luciferase-expressing ovarian cancer 43, 163 and was found to induce destruction of a large fraction of the tumor cells (NIR-PIT vs. control at day 7, *P* = 0.0037) 43. Galactosyl serum albumin (GSA) is a surface lectin that can bind weakly to beta-D-galactose receptors overexpressed in ovarian cancer 164. Although GAS-mediated NIR-PIT has been evaluated in disseminated peritoneal ovarian cancer, it appears to be less potent than antibody-mediated therapy. Two possible reasons for this might be the weak binding affinity of GSA to cancer cell membranes and the massive internalization of GSA-IR700 by cells. However, when no specific antibody is available, GSA may be a useful alternative target molecule. Moreover, repeated anti-GSA NIR-PIT treatment could help improve tumor suppression by promoting deeper penetration of GSA-IR700 into tumors following the initial treatment 163.

Integrins are transmembrane cell adhesion proteins that can act as signaling receptor proteins to regulate cancer progression by binding to extracellular matrix (ECM) components. Additionally, some integrins are overexpressed in ovarian cancer, neovasculature, and on immunosuppressive lymphocytes 165. Approximately half of integrins bind to the ECM and cytoskeleton by recognizing the RGD sequence. Thus, RGD-containing peptides are used as carriers for targeting cancers 166. In fact, an RGD-based NIR-PIT targeting integrins exhibits strong phototoxicity in a 3D model of ovarian cancer cells 79. Since immunosuppressive lymphocytes contribute to a poor prognosis, NIR-PIT might further improve the therapeutic response by eliminating these immunosuppressive cells within the TME 167.

### Pancreatic cancer

Pancreatic cancer cells express high levels of CEA 168. CEA-targeted NIR-PIT induces extensive cell death and a significant reduction in tumor size and weight in an orthotopic mouse xenograft model. However, the tumor eventually recurred in all treated mice, likely due to the administration only a single round of treatment 169. CEA-mediated NIR-PIT has also been used as a postsurgical treatment for the prevention of local and metastatic recurrence. In an orthotopic mouse model with a human pancreatic cancer cell line, bright light surgery (BLS) plus NIR-PIT decreased the local and metastatic recurrence rate to 1/7 mice (*P* = 0.001) and 2/7 mice (*P* = 0.03), compared with 7/7 mice and 6/7 mice respectively, in the BLS alone group. Significant differences in the tumor growth rate, tumor size, and tumor weight between the two groups persisted even several weeks after surgery 57. NIR-PIT was also effective when applied in a patient-derived orthotopic xenograft (PDOX) mouse model. The local recurrence rate was reduced from 85.7% in the BLS group to 28.6% in the BLS and NIR-PIT combination group (*P* = 0.05). Moreover, the average weight of recurrent tumors was significantly lower in BLS+PIT-treated mice than in the BLS alone group (*P* = 0.015) 170.

Cadherin-17 (CDH17 or CA17), a calcium-dependent transmembrane glycoprotein, is highly expressed in many gastrointestinal cancers including pancreatic adenocarcinoma. CDH17-targeted NIR-PIT significantly inhibits tumor growth in a pancreatic adenocarcinoma xenograft model after three cycles of treatment 171.

### Prostate cancer (PCa)

Prostate-specific membrane antigen (PSMA) is an attractive target for the treatment of PCa using NIR-PIT as it is highly expressed in prostate cancer cells and in the neovasculature. PCa can be illuminated with NIR light either directly or via a transurethral catheter 62. Different tumor-homing ligands have been used to construct TSPCs for PCa treatment, including antibodies, Db, Mb, and small-molecule agents. Whole IgG-mediated NIR-PIT has been shown to strongly inhibit tumor growth and improve overall survival in a xenograft mouse model 62, 63, 89. Meanwhile, Db, Mb, and whole IgG-based TSPCs display similar antitumor effects at the same molar concentration. However, the smaller Dbs allow the shortest interval between TSPC injection and light exposure, which may have advantages in the clinical translation of photoimmunotherapy 89. Moreover, a PMSA-targeted TSPC, i.e., YC-9, was constructed by conjugating IR700 to the PMSA-binding small molecule Lys-Glu-urea through a lysine-suberate linker. YC-9 exhibits PSMA-specific cell killing, resulting in delayed tumor growth 61. Moreover, to apply NIR-PIT in surgery, Derks et al. developed a novel agent by incorporating radionuclide indium-111 into Lys-Glu-urea-IR700 TSPC to optimize the multimodal image-guided PCa surgery via combination with targeted NIR-PIT. This novel design could assist in locating both distant tumor lesions and metastatic lymph nodes, while also facilitating the removal of residual tumors without damaging important structures, such as the nerves controlling the urinary bladder 94.

## NIR-PIT clinical studies/trials in humans

The potential efficacy and safety of NIR-PIT make it a promising modality for cancer therapy, leading to its evaluation in various clinical trials worldwide. More specifically, in the United States (US), a phase I/IIa clinical trial of NIR-PIT based on a conjugate comprising cetuximab and IR700 (cetuximab-IR700, RM-1929) has recently concluded. Results indicate that a manageable safety profile and substantial antitumor responses were obtained in locoregional, recurrent HNSCC patients who had previously exhibited unsatisfactory therapeutic responses to traditional clinical therapy 109. In Japan, a phase I, open‑label, single‑center study of NIR-PIT (RM‑1929) in patients with recurrent HNSCC showed a similar safety profile and promising antitumor activity to that observed in the American phase I/IIa study 110. Given these clinically meaningful results, a global phase III trial including 275 patients with recurrent or second primary HNSCC is currently underway (NCT03769506). In September 2020, cetuximab-IR700 was approved by the Japanese government for the treatment of unresectable locally advanced or recurrent HNSCC 172. In December 2020, an open-label, phase I/II tumor immunotherapy trial was initiated using RM‑1929-mediated NIR-PIT in combination with anti-programmed cell death protein 1 (PD1) immune checkpoint inhibitors in patients with recurrent or metastatic HNSCC, or advanced or metastatic CSCC (NCT04305795). Furthermore, based on encouraging results in animal models, a phase Ib/II clinical trial is currently underway in the US to investigate the safety, immunogenicity, and efficacy of AU-011-mediated NIR-PIT in patients with small primary choroidal melanomas (NCT03052127).

## NIR-PIT for non-malignant conditions

### Infection

The increasing prevalence of antimicrobial-resistant bacteria necessitates the development of alternative antimicrobial strategies, including targeted antimicrobial photodynamic therapy (aPDT). Bispo et al. used an antibody against the immunodominant staphylococcal antigen A (IsaA) for IR700 conjugation, which successfully targets and destroys methicillin-resistant *Staphylococcus aureus* (MRSA) cells after light irradiation. With the help of the enhancing agent potassium iodide, the anti-IsaA NIR-PIT can eliminate a high MRSA titer, regardless of the strong antioxidant activity of plasma 173. Moreover, Yasui et al. used low-cost polyclonal chicken egg yolk IgY antibodies for the construction of a TSPC to kill *Candida albicans*. In a cutaneous infected mouse ulcer model, TSPC led to skin recovery after NIR irradiation with minimal damage to the uninfected epithelium 174. Reessing et al. described a general strategy for the conjugation of IRdye800CW/IR700 with the small-molecule antibiotics vancomycin and amphotericin B. Vancomycin-IRdye800CW conjugates can specifically bind to gram-positive bacteria, not gram-negative bacteria. The vancomycin-IR700 conjugates retain the ability of IR700 to produce reactive oxygen species, facilitating targeted action against invading pathogens 175. Meanwhile, as an antiviral treatment, Sadraeian et al. used NIR-PIT to kill HIV-infected cells by targeting the Gp41 glycoprotein, a component of the HIV envelope spike (Env). This anti-Gp41 therapy elicits cytotoxicity in HIV-env-expressing cells 176. However, in general, microbial growth is greater than that of cancer cells. In this regard, the amount of TSPC and the NIR light energy may need to be increased to achieve better therapeutic efficacy.

### Pain

Innocuous touching provokes severe pain in patients with mechanical allodynia, a major symptom of neuropathic pain. Peripheral sensory neurons expressing receptor tyrosine kinase B (TrkB) can sense the lightest touch under basal conditions and have been identified as necessary for mechanical allodynia in mice after nerve injury. In models of neuropathic pain, brain-derived neurotrophic factor (BDNF, a ligand for TrkB) selectively mediated NIR-PIT and effectively eliminated TrkB-positive sensory fibers, thus reversing mechanical allodynia in multiple types of neuropathic pain 177. Another attractive target for reducing pain is nerve growth factor (NGF) as this ligand, and its receptor TrkA/p75, play critical roles in the development and function of peripheral nociceptive neurons 178. Accordingly, NGF derivatives conjugated with IR700 selectively photoablate TrkA-positive nociceptors and substantially reduce the response to point-based nociceptive mechanical stimuli after irradiation of the injected area. Moreover, photoablation using NGF derivatives also abolishes long-term mechanical hypersensitivity in mouse models of inflammatory, osteoarthritic, and neuropathic pain. Notably, the behavioral responses to dynamic mechanical stimuli are not altered by NGF-mediated photoablation, as these dynamic stimuli are transduced by TrkB-positive mechanoreceptors after nerve injury 179.

In summary, NIR-PIT can directly attack cells to bypass the complex signaling pathways underlying pain responses. Moreover, the TSPC dosage, light intensity, and frequency of application are adjustable, thus allowing for personalized therapeutic regimens. Indeed, the long-lasting efficacy of a single photoablation treatment regimen has the potential to reduce toxicity compared with other therapies.

### Itch

Chronic itch causes an urgent desire for scratching and subsequent skin damage, which is not only difficult to treat, but also impacts the quality of life to the same extent as pain 180. The cytokine interleukin (IL)-31 has been identified as a key trigger in mediating the itch response 181. In fact, a single treatment with IL-31 receptor-targeted NIR-PIT can induce long-lasting relief of itch-associated behavior in mouse models of atopic dermatitis and familial primary localized cutaneous amyloidosis (FPLCA), thus facilitating skin healing. The advantages and underlying mechanisms of NIR-PIT for treating itch are similar to those summarized above for pain; the mechanism is reportedly associated with the retraction of itch-sensing neurons from the epidermis 182.

### Hyperinsulinemic hypoglycemia

Uncontrolled production of insulin by pancreatic β-cells can cause hyperinsulinemic hypoglycemia 183. The GLP-1 receptor (GLP-1R) is expressed in pancreatic β-cells and in virtually all benign insulinomas 184. As expected, NIR-PIT employing an analog of GLP-1 can specifically bind and kill GLP-1R-positive cells, providing a new, effective, and minimally invasive approach to normalize blood glucose levels in patients with hyperinsulinemic hypoglycemia 185.

### Rheumatoid arthritis (RA)

Fibroblast-like synoviocytes (SFs) are a unique cell type that distinguishes RA from other inflammatory diseases affecting the joints 186. FAP is a surface marker for activated SFs. The upregulation of FAP on activated SFs reportedly correlates with the long-term progression of RA 187. Dorst et al. applied NIR-PIT to target FAP in a mouse model of RA and reported that the development of arthritis was delayed by selectively depleting FAP-positive fibroblasts in mice with collagen-induced arthritis 188. In addition, FAP-based NIR-PIT was found to kill FAP-positive fibroblasts within a population of primary synovial fibroblasts, and biopsies from human RA synovial tissue. 189. This novel FAP-mediated approach may have potential for treating RA, as an alternative to immunosuppressive drugs.

### Systemic sclerosis (SSc)

SSc, also called scleroderma, is a complex autoimmune disease characterized by inflammation, vasculopathy, and fibrosis of the skin and internal organs. This fibrosis is partially caused by the presence of activated myofibroblasts (myo) in the tissues 190. Similar to RA, FAP is specifically expressed on activated fibroblasts in SSc; thus, FAP-targeted NIR-PIT has the potential to eliminate pathological fibroblasts in SSc skin. In fact, FAP-mediated NIR-PIT could deplete primary skin fibroblasts from SSc patients in 2D/3D cultures and fully prevented the contraction of SSc fibroblasts in 3D cultures, which is critical for tissue stiffness and fibrosis 191.

### Elimination of target cells from mixed cell cultures or tissues

It is desirable, yet difficult, to eliminate a specific type of cell from mixed cell cultures or tissues without damaging adjacent cells. By employing targeted antibodies and spatially selective irradiation, NIR-PIT could eliminate specific cells from mixed 2D/3D cell cultures or tissues* ex vivo,* and from mixed populations in tumor models without damaging non-targeted cells 192, 193. This technique could remove potential interference and contamination by unwanted cells.

### Limitations and challenges of NIR-PIT

Despite the very promising therapeutic effects, a few potential limitations and challenges remain when considering NIR-PIT implementation. First, light exposure can cause potential toxicity to organs located in proximity to the tumor. In fact, mild peri-tumor dermal edema is one of the most common treatment emergent adverse events (TEAE) reported in patients treated with NIR-PIT. Meanwhile, low-grade, reversible skin photosensitivity was observed in a small proportion of treated patients 109, 110. Moreover, exposure of liver or kidney metabolic organs to light might also lead to damage. Second, as the penetration light depth is limited and illumination is localized, it is difficult for NIR-PIT to treat all disseminated sites or those located in deep organs or tissues where the light irradiation is not accessible. However, since IR700 emits near-infrared fluorescence, optical imaging can help to map and irradiate the primary and metastatic tumors. Meanwhile, NIR-PIT using an endoscopic fiber optic diffuser is a promising candidate for the treatment of disseminated peritoneal metastases 54, 55. Moreover, the combination of NIR-PIT and immune-checkpoint inhibitors/cytokines might prove effective for the treatment of distant tumors by enhancing antitumor immunity 8. Notably, a majority of preclinical studies performed using immunodeficient mice, such as athymic nude mice, which prevented the ICD effects and a more robust anti-tumor immune response when combined with immunomodulators. However, in mice with intact immune systems, in addition to the local antitumor immune response, NIR-PIT might induce systemic cytokine storms, which may lead to potential adverse effects 84. Third, issues have been reported regarding the selection of target molecules, development of tumor lysis syndrome, and the high-cost of TSPCs. However, potential solutions are being investigated, and new strategies and techniques are being developed 22, 23.

## Conclusions

NIR-PIT has been widely studied in various types of cancer, and noncancerous diseases, both *in vitro* and *in vivo* animal models, showing promising therapeutic effects and leading to its application in clinical trials. A wide range of targets can be selected for binding by NIR-PIT agents. Often, tumor-associated cell-surface antigens or glycoproteins, such as EGFR, HER2, or PSMA, are used as tumor-homing targets, given that they are overexpressed on many tumors. In the TME, targets expressed on the non-tumoral cells that support and maintain the tumor have also been investigated. Moreover, multiple antigens can be targeted simultaneously using a cocktail of TSPCs in an NIR-PIT setting.

Antigen-specific recognition agents, including antibodies and their fragments, peptides, protein ligands, and small molecules have been employed in the construction of TSPCs. Each has different advantages and can be selected depending on the function and cellular distribution of the antigen.

NIR light is also a critical factor in NIR-PIT. Light dose, power density, and source influence the therapeutic efficacy. Light delivery systems are of great importance for NIR-PIT. Although NIR light is most commonly delivered by an external light source, endoscopy and wireless implantable light systems have also been used. NIR-PIT, as an adjunctive therapy, could eliminate residual tumor cells after BLS, which is also feasible to reduce metastasis and local recurrence. NIR-PIT can be combined with other modalities to achieve improved antitumor effects. The advantages of these combinations can be ascribed to SUPR effects and other synergistic antitumor mechanisms, such as enhancing antitumor immunity by combination with an immune checkpoint blockade. Recent studies have reported only minimal adverse events associated with NIR-PIT. However, the safe range of TSPC doses and light doses should be further explored. Overall, NIR-PIT holds great promise for clinical applications to treat cancer and other non-malignant diseases with targetable antigens/receptors. However, the toxicity and safety of NIR-PIT must be fully investigated before it can be translated into clinical settings.

## Figures and Tables

**Table 1:** Therapeutic targets on cancer cells.

Target molecule	IR700 carrier	TSPC	Cancer	Combination treatment	Tumor model	Ref.
AC133/CD133	Antibody	AC133-IR700	Glioblastoma		*In vitro and in vivo;* ORT	74
ꞵ-D-galactose receptor	Ligand	GSA-IR700	Peritoneal disseminated ovarian cancer		*In vitro and in vivo;* ORT	163
Cadherin-17	Antibody	ARB102-IR700	Pancreatic cancer		*In vitro and in vivo;* s.c.	171
CB_2_R	Ligand	mbc94-IR700	Brain cancer		*In vitro and in vivo;* s.c.	118, 119
CD146	Antibody	YY146-IR700	Melanoma		*In vitro and in vivo;* s.c.	67
CD20	Antibody	Rituximab-IR700	B-cell lymphoma		*In vitro and in vivo;* s.c.	65
CD20	Antibody	NuB2-IR700	B-cell lymphoma		*In vitro and in vivo;* s.c.	66
CD29	Antibody	Anti-CD29-IR700	Melanoma	CTLA4 blockade	*In vitro and in vivo;* s.c.	159
CD276	Fab fragment of antibody	αCD276/Fab-IR700	Breast cancer	αPD-L1 antibody	*In vitro and in vivo;* s.c. and lung metastasis	90
CD44	Antibody	Anti-CD44-IR700	Triple-negative breast cancer		*In vitro and in vivo;* ORT	72
CD44	Antibody	Anti-CD44-IR700	Oral squamous cell carcinoma		*In vitro and in vivo;* s.c.	73
CD44	Antibody	Anti-CD44-IR700	Colon cancer, lung carcinoma, oral carcinoma	PD-1 blockade	*In vitro and in vivo;* s.c.	102
CD44	Antibody	Anti-CD44-mAb-IR700	Colon cancer, lung carcinoma, oral carcinoma	CTLA4 blockade	*In vitro and in vivo;* s.c.	103
CD44	Antibody	anti-CD44-mAb-IR700	Colon cancer, lung carcinoma, oral carcinoma	IL-15	*In vitro and in vivo;* s.c.	104
CD47	Antibody	B6H12-IR700	Bladder cancer		*In vitro and in vivo;* s.c.	75
CEA	Antibody	45IR (C2-45 IR700)	Gastric adenocarcin-oma		*In vitro and in vivo;* s.c.	12
CEA	Antibody	Anti-CEA-IR700	pancreatic cancer		*In vitro and in vivo;* ORT	169
CEA	Antibody	Anti-CEA-IR700	After bright light surgery in mouse models of pancreatic cancer		*In vitro and in vivo;* ORT	57
CEA	Antibody	Anti-CEA-IR700	After bright light surgery in PDOX model of pancreatic cancer		*In vitro and in vivo;* ORT	170
CEA	Antibody	DTPA-hMN-14-IR700	Colorectal cancer		*In vitro and in vivo;* s.c.	136
CEA	Antibody	M5A-700	Colorectal cancer		*In vitro and in vivo;* ORT	58
c-KIT	Antibody	12A8-IR700	Gastrointest-inal stromal tumors		*In vitro and in vivo;* ORT	148
CLA	Antibody	CLA-IR700DX	Mycosis fungoides		*In vitro*	161
DLL3	Antibody	Rova-IR700	Lung cancer		*In vitro and in vivo;* s.c.	151
EGFR	Antibody	Cet-IR700	Head and neck cancer		*In vivo*/clinical trial	109, 110
EGFR	Antibody	Pan-IR700	Head and neck cancer		*In vitro and in vivo;* s.c. (subtotal tumor resection model)	34
EGFR	Antibody	Pan-IR700	Bladder cancer		*In vitro and in vivo;* s.c.	36
EGFR	Antibody	Can225-IR700	Bladder cancer		*In vitro and in vivo;* s.c.	38
EGFR	Antibody	Cet-IR700 or pan-IR700	Breast cancer, cutaneous squamous cell carcinoma		*In vitro and in vivo;* ORT	97
EGFR	Antibody	Cet-IR700	Triple-negative breast cancer		*In vitro and in vivo;* s.c.	46
EGFR	Antibody	Pan-IR700	Breast cancer		*In vitro and in vivo;* ORT	50, 100
EGFR	Antibody	Pan-IR700	Breast cancer	CyEt-Pan-Duo	*In vitro and in vivo;* s.c.	47
EGFR	Antibody	Pan-IR700	Lung cancer		*In vitro and in vivo;* ORT	40
EGFR	Nanobody	Nanobody-IR700 (7D12, 7D12-9G8)	Head and neck cancer		*In vitro and in vivo;* ORT	35
EGFR	Affibody	Z_EGFR:03115_-IR700	Glioblastoma		*In vitro and in vivo;* s.c.	33
EGFR	Affibody	ZEGFR:1907-IR700	Multidrug resistant colorectal cancer		*In vitro and in vivo;* s.c.	141
GPC1	Antibody	Miltuximab®-IR700	Bladder, prostate, brain,ovarian cancer cell lines		*In vitro*	130
GPC3	Antibody and heavy-chain antibody	HN3-IR700, YP7-IR700	Hepatocellu-lar carcinoma		*In vitro and in vivo;* s.c.	59
GPC3	Antibody	YP7-IR700	Hepatocellu-lar carcinoma	Nanosized albumin-bound paclitaxel	*In vitro and in vivo;* s.c.	60
GPA33	ScFv	A33scFv-R700	Colorectal tumors		*In vitro and in vivo;* s.c.	138
HER2	Antibody	Tra-IR700	Lung metastases		*In vitro and in vivo;* ORT and lung metastasis	39, 41
HER2	Antibody	Tra-IR700	Pleural disseminated non-small cell lung carcinoma		*In vitro and in vivo;* ORT	42
HER2	Antibody	Tra-IR700	Disseminated peritoneal ovarian cancer		*In vitro and in vivo;* ORT	43
HER2	Antibody	Tra-IR700 and Per-IR700	Gastric cancer		*In vitro and in vivo;* s.c.	53
HER2	Antibody	Tra-IR700	Peritoneal dissemination of gastric cancer		*In vitro and in vivo;* ORT	54, 55
HER2	Antibody	Tra-IR700	Gastric cancer	5-FU	*In vitro and in vivo;* s.c.	52
HER2	Nanobody	1D5-IR700, 1D5-18A12-IR700	Breast cancer		*In vitro and in vivo;* ORT	88
HER2	Affibody	HER2 Affibody-IR700	Breast Cancer		*In vitro*	124
HER2	Affibody	Z_HER2:2395_-IR700	Ovarian cancer		*In vitro and in vivo;* s.c.	93
HGFR	Nanobody	G2c-PS	Hepatocellu-lar, gastric cancer cell lines		*In vitro*	91
HSPG	VLP	AU-011 (IR700)	Primary uveal melanoma		*In vitro and in vivo;* s.c. (murine) and ORT (rabbit)	95
Mesothelin	Antibody	HYP218-IR700	Cutaneous squamous cell carcinoma stably expressing human mesothelin		*In vitro and in vivo;* s.c.	142
PSMA	Antibody, Diabody, Minibody	PSMA-Db-IR700, PSMA-Mb-IR700, PSMA-IgG-IR700	Prostate cancer		*In vitro and in vivo;* s.c.	89
PSMA	Antibody	^111^ In-DTPA-D2B-IR700	Prostate cancer		*In vitro and in vivo;* s.c.	63
PSMA	Antibody	Anti-PSMA- IR700	Prostate cancer		*In vitro and in vivo;* s.c.	62
PSMA	Peptide	PSMA-1-Pc413, PSMA-1-IR700	Prostate cancer		*In vitro and in vivo;* s.c.	64
PSMA	Small molecule	YC-9	Prostate cancer		*In vitro and in vivo;* s.c.	61
PD-1	Antibody	Avelumab-IR700	Papillary adenocarci-noma of lung		*In vitro and in vivo;* s.c.	154
PDPN	Antibody	NZ-1-IR700	Malignant pleural mesothelioma		*In vitro and in vivo;* s.c. and disseminated pleural models	155
Pgp	Antibody	Pab-IR700	Multidrug-resistant cervical cancer		*In vitro and in vivo;* s.c.	69
Pgp	Antibody, Fab fragment	Fab-IR700, Pab-IR700	Multidrug-resistant cervical cancer		*In vitro and in vivo;* s.c.	70
TROP2	Antibody	TROP2-IR700	Biliary & pancreatic cancer		*In vitro and in vivo;* s.c.	134
US28	Nanobody	VUN100-IR700	Glioblastoma		*In vitro* (2D and 3D cultures)	92

CEA: carcinoembryonic antigen; GSA: galactosyl serum albumin; HGFR: hepatocyte growth factor receptor, c-Met; PDOX: patient-derived orthotopic xenograft; TSPC: target-specific photosensitizer conjugate; VLP: virus-like particle; s.c, subcutaneous; ORT, orthotopic

**Table 2:** Therapeutic targets on non-tumor cells in the tumor microenvironment (TME).

Target molecule	IR700 carrier	TSPC	Cancer	Combination treatment	Tumor model	Ref.
CD25	Antibody	Anti-CD25-IgG-IR700, Anti-CD25-F(ab′)2-IR700	Colon cancer		*In vitro and in vivo;* s.c.	82
CD25	F(ab')_2_ fragments	Anti-CD25-F(ab')_2_-IR700	Colon cancer, lung carcinoma, Prostate cancer		*In vitro and in vivo;* s.c.	84
CD73	Antibody	αCD73-IR700	Pancreatic ductal adenocarcinoma(subcutaneous), breast cancer (orthotopic)	PD-1 blockade	*In vitro and in vivo;* s.c. and ORT	77
FAP	Antibody	FAP-IR700	Esophageal squamous cell carcinoma		*In vitro and in vivo;* s.c.	76
FAP	Antibody	FAP-IR700	Esophageal cancer	5-FU	*In vitro and in vivo;* s.c.	146
Integrin α_v_β_3_	Nanobody	cRGD-PEG-HSA-IR700	Ovarian tumor spheroids		*In vitro* (in 3D cultures)	79
Integrin α_v_β_3_	Nanobody	IR700-PEG-PGlu-cRGDx	Glioblastoma		*In vitro and in vivo;* s.c.	80
Integrin α_v_β_3_	Nanobody	RGD-8PEG-IR700	Melanoma spheroids		*In vitro* (in 3D cultures)	81
PDGFRβ	Affibody	Dimeric Z-PDGFRβ-IR700	Colorectal cancer		*In vitro and in vivo;* s.c.	78
VEGFR-2	Antibody	DC101-IR700	Gastric cancer		*In vitro and in vivo;* s.c.	56
VISTA	Antibody	anti-VISTA-IR700	Colon cancer, lung carcinoma		*In vitro and in vivo;* s.c.	85

s.c, subcutaneous; ORT, orthotopic

**Table 3:** Combined targeting of multiple targets or epitopes.

Target molecule	IR700 carrier	TSPC	Cancer	Combination treatment	Tumor model	Ref.
EGFR, HER2	Antibody	Pan-IR700, Tra-IR700	Cholangiocarci-noma		*In vitro and in vivo;* s.c.	135
EGFR, HER2	Antibody	Pan-IR700, Tra-IR700	Bladder cancer		*In vitro and in vivo;* s.c.	86
EGFR, HER2	Antibody	Cet-IR700, Tra-IR700	Esophageal adenocarcinoma	TKI	*In vitro*	51
EGFR, HER2	Antibody	Cet-IR700, Tra-IR700	Cutaneous squamous cell carcinoma		*In vitro and in vivo;* s.c.	20
EGFR, CD25	Antibody	Pan-IR700, Bas-IR700	Cutaneous squamous cell carcinoma		*In vitro and in vivo;* s.c.	27
EGFR, EpCAM and CSPG4	ScFv	ScFv-425 (EGFR), EpCAM(scFv), CSPG4(scFv), -SNAP-IR700	Triple-negative breast cancer cell lines		*In vitro*	49
CD44, CD25	Antibody	Anti-CD44-IR700, anti-CD25-IR700	Colon cancer, lung carcinoma, oral carcinoma		*In vitro and in vivo;* s.c.	83

TKI: tyrosine kinase inhibitor; s.c, subcutaneous

**Table 4:** NIR-PIT for non-malignant diseases.

Target molecule	IR700 carrier	TSPC	Noncancerous diseases	Model	Ref.
FAP	Antibody	28H1-700DX	Arthritis	*In vitro* and *in vivo* collagen-induced arthritis mouse model	188
FAP	Antibody	28H1-IR700	Systemic sclerosis	*In vitro* (2D and 3D cultures)	191
GLP-1R	Ligand, peptide	Exendin-4-IR700	Hyperinsulinemic hypoglycemia	*In vitro* and *in vivo*; s.c.	185
Gp41	Antibody	7B2-IR700	HIV	*In vitro*	176
IgY	Antibody	CA-IgY-IR700	*Candida albicans* infection	*In vitro* and *in vivo* CA-infected cutaneous ulcers mouse model	174
IL-31	Ligand	IL-31^K138A-SNAP^-IR700	Itch	*In vitro* and *in vivo* mouse models of atopic dermatitis, of cutaneous amyloidosis, and of psoriasis	182
IsaA	Antibody	1D9-IR700	*Staphylococcus aureus* infections	*In vitro* and *in vivo Galleria mellonella* larval infection model, human postmortem orthopedic implant infection model	173
TrkA/ P75	Ligand	NGF^R121W-SNAP^-IR700	Acute, inflammatory, neuropathic, and joint pain	*In vitro* and *in vivo* mouse models of pain	179
TrkB	Ligand	BDNF^SNAP^-IR700	Mechanical pain hypersensitivity	*In vitro* and *in vivo* mouse pain models	177

s.c, subcutaneous

## Abbreviations

ADC: antibody-drug conjugate
ADCC: antibody-dependent cell-mediated cytotoxicity
aPDT: antimicrobial photodynamic therapy
BL: B cell lymphoma
BLS: bright light surgery
CAF: cancer-associated fibroblast
CCA: cholangiocarcinoma
CEA: carcinoembryonic antigen
CR: complete remission
CRC: colorectal cancer
CSC: cancer stem cell
CSCC: cutaneous squamous cell carcinoma
DAMP: damage-associated molecular pattern
EC: esophageal cancer
ECM: extracellular matrix
FPLCA: familial primary localized cutaneous amyloidosis
GBM: glioblastoma
GIST: gastrointestinal stromal tumor
GPCR: G protein-coupled receptors
GSA: galactosyl serum albumin
HCC: hepatocellular carcinoma
HNSCC: head and neck squamous cell carcinoma
ICD: immunogenic cell death
MDR: multidrug resistant
MDSC: myeloid-derived suppressor cell
MF: mycosis fungoides
MPM: malignant pleural mesothelioma
NHS: N-hydroxysuccinimide ester
NIR-PIT: near-infrared photoimmunotherapy
PC: pancreatic carcinoma
PCa: prostate cancer
PDOX: patient-derived orthotopic xenograft
PDT: photodynamic therapy
PS: photosensitizer
RA: rheumatoid arthritis
SCCHN: squamous cell carcinoma of the head and neck
SCLC: small cell lung cancer
SF: synoviocyte
SSc: systemic sclerosis
SUPR: superenhanced permeability and retention
TAA: tumor-associated antigen
TKI: tyrosine kinase inhibitor
TME: tumor microenvironment
TNBC: triple-negative breast cancer
TSPC: target-specific photosensitizer conjugate
VLP: virus-like particle
